# Cognitive Processes Associated With Sudden Gains in Cognitive Therapy for Posttraumatic Stress Disorder in Routine Care

**DOI:** 10.1037/ccp0000488

**Published:** 2020-03-05

**Authors:** Milan Wiedemann, Richard Stott, Alecia Nickless, Esther T. Beierl, Jennifer Wild, Emma Warnock-Parkes, Nick Grey, David M. Clark, Anke Ehlers

**Affiliations:** 1 Department of Experimental Psychology, University of Oxford, and Oxford Health NHS Foundation Trust, Oxford, United Kingdom; 2 Department of Psychology, King’s College London, and National Institute for Health Research Mental Health Biomedical Research Centre, South London Maudsley NHS Foundation Trust, London, United Kingdom; 3 Nuffield Department of Primary Care Health Sciences, University of Oxford, and Oxford Health NHS Foundation Trust, Oxford, United Kingdom; 4 Department of Experimental Psychology, University of Oxford; 5 Department of Experimental Psychology, University of Oxford, and Oxford Health NHS Foundation Trust, Oxford, United Kingdom; 6 Department of Experimental Psychology, University of Oxford, and Department of Psychology, King’s College London; 7 Department of Psychology, King’s College London, and National Institute for Health Research Mental Health Biomedical Research Centre, South London Maudsley NHS Foundation Trust, London, United Kingdom; 8 Department of Experimental Psychology, University of Oxford, and Oxford Health NHS Foundation Trust, Oxford, United Kingdom

**Keywords:** posttraumatic stress disorder, cognitive therapy, sudden gains, mechanisms of change, cognitions

## Abstract

***Objective:*** Although most studies investigating sudden gains in treatments for posttraumatic stress disorder (PTSD) report a positive association between sudden gains and outcomes at the end of treatment, less is known about sudden gains in routine clinical care and the processes involved in their occurrence. This study investigated changes in cognitive factors (negative appraisals, trauma memory characteristics) before, during, and after sudden gains in PTSD symptom severity. ***Method:*** Two samples (*N*_1_ = 248, *N*_2_ = 234) of patients who received trauma-focused cognitive therapy for PTSD in routine clinical care were analyzed. Mahalanobis distance matching, including the propensity score, was used to compare patients with sudden gains and similar patients without sudden gains. Estimates from both samples were meta-analyzed to obtain pooled effects. ***Results:*** Patients with sudden gains (*n*_1_ = 76, *n*_2_ = 87) reported better treatment outcomes in PTSD symptom severity, depression, and anxiety at the end of therapy and follow-up than those without sudden gains. No baseline predictors of sudden gains could be reliably identified. During sudden gains, those with sudden gains had greater changes in both cognitive factors than matched patients. Meta-analyses of the two samples showed that negative appraisals had already decreased in the session prior to sudden gains compared with matched patients. ***Conclusions:*** The pooled estimates suggest that changes in negative trauma-related appraisals precede sudden gains in PTSD symptoms. The results suggest that interventions that promote change in appraisals may also facilitate sudden gains in therapy.

## What is the public health significance of this article?

This study highlights that a substantial subgroup of patients with PTSD showed concurrent large improvements in PTSD, appraisals, and memory features from one treatment session to the next. Sudden gains were also preceded by greater changes in appraisals than sessions that did not include sudden gains. This supports interventions that target the identification and modification of negative appraisals in PTSD, which, if successful, can result in sudden symptom improvements that are associated with better treatment outcomes.

Sudden gains are large and stable symptom improvements experienced by a patient from one therapy session to the next. Tang and DeRubeis (1999) developed three criteria to identify sudden gains: The gain should be large in absolute magnitude, large relative to the previous symptom score, and large relative to symptom fluctuation. The authors applied these criteria to a sample of 61 patients who received cognitive–behavioral therapy for depression and found that the 24 patients (39%) who experienced a sudden gain reported better outcomes at the end of treatment and at follow-up compared with all other patients who did not experience a sudden gain. Further, coding of video recordings of the sessions by independent raters showed that patients with sudden gains showed a greater shift in cognitions during the session immediately before the sudden gain in comparison to a control session within the same patients. Tang and DeRubeis (1999) found that patients reported an increase in therapeutic alliance immediately after the sudden gain and hypothesized that sudden gains lead to a better therapeutic alliance, which enables further improvements during therapy.

Several studies have replicated and expanded upon Tang and DeRubeis’s (1999) findings and methods in different psychological treatments and disorders, primarily analyzing data from randomized controlled trials, for example, posttraumatic stress disorder (PTSD; e.g., Kelly, Rizvi, Monson, & Resick, 2009), generalized anxiety disorder (e.g., Deschênes & Dugas, 2013), social anxiety disorder (e.g., Hofmann, Schulz, Meuret, Moscovitch, & Suvak, 2006), panic disorder (e.g., Clerkin, Teachman, & Smith-Janik, 2008), and obsessive–compulsive disorder (e.g., Aderka et al., 2012). The positive relationship between sudden gains and better outcomes at the end of therapy was replicated in treatments for depression, anxiety, and PTSD (for a review, see Aderka, Nickerson, Bøe, & Hofmann, 2012).

Similarly, six studies found that sudden gains in PTSD symptoms are linked to better treatment outcomes at the end of psychological therapy for PTSD (Aderka, Appelbaum-Namdar, Shafran, & Gilboa-Schechtman, 2011; Doane, Feeny, & Zoellner, 2010; Jun, Zoellner, & Feeny, 2013; Kelly et al., 2009; König, Karl, Rosner, & Butollo, 2014; Krüger et al., 2014), and only one study found no such association (Haugen, Goldman, & Owen, 2015). Out of the six studies reporting an association between sudden gains and posttreatment outcomes, two studies also reported an association between sudden gains and better outcomes at follow-up (Aderka et al., 2011; Kelly et al., 2009), two reported no effect on follow-up measures (König et al., 2014; Krüger et al., 2014), and two did not investigate this question (Doane et al., 2010; Jun et al., 2013). To our knowledge, only two studies with relatively small samples (*n* = 63 and 26) investigated sudden gains in treatments for PTSD in routine clinical care (Aderka et al., 2011; Doane et al., 2010). Further research in larger routine clinical care samples is needed to evaluate how common sudden gains are in routine clinical settings and how they are related to outcome. Furthermore, an important question is what processes of change contribute to sudden gains. It has not as yet been examined how changes in cognitive factors thought to contribute to the maintenance of PTSD (e.g., negative appraisals or trauma memory characteristics, Brewin, 2014; Ehlers & Clark, 2000; Foa & Riggs, 1993; Resick & Schnicke, 1993) are associated with sudden gains in PTSD symptoms. These theories would predict that changes in these cognitive factors not only accompany sudden gains in symptoms but also predict them because changes in cognitions are thought to drive symptom change.

Change processes associated with sudden gains have primarily been investigated in studies in treatments for depression and social anxiety. Support for the hypothesis that cognitive change precedes sudden gains was found in some depression studies (e.g., Tang & DeRubeis, 1999; Tang, DeRubeis, Beberman, & Pham, 2005), whereas other studies of patients with depression or social anxiety did not find such an association (e.g., Andrusyna, Luborsky, Pham, & Tang, 2006; Bohn, Aderka, Schreiber, Stangier, & Hofmann, 2013; Hofmann et al., 2006; Vincent & Norton, 2019). Reasons for the reported discrepancies in change processes associated with sudden gains in depression may partly be due to differences in the clinical samples and treatments. Replications of sudden-gains studies in comparable clinical samples are scarce (cf., Wucherpfennig, Rubel, Hollon, & Lutz, 2017). Further, the methods used to address the question of which processes are associated with sudden gains differ in the time points at which the process variables were measured (e.g., baseline differences, between-session changes immediately prior to the sudden gain, or within-session changes in the pregain session) and the methods to select a comparison group (within-patient comparisons, between-patient comparisons, or both). Although these methods aim to answer a similar research question, the differences are likely to influence the results and complicate the comparison between studies (Vincent & Norton, 2019; Wucherpfennig, Rubel, Hofmann, & Lutz, 2017).

In addition to testing processes and predictors preceding sudden gains, recent studies have also investigated processes following sudden gains. Wucherpfennig, Rubel, Hofmann, et al. (2017) replicated Tang and DeRubeis’s (1999) findings that sudden gains in depression lead to an improvement in the therapeutic alliance and further found that patients reported an increase in coping skills following sudden gains. Further research investigating how other clinically relevant factors change following sudden gains may help understand the processes of change.

To our knowledge, no study has yet investigated the cognitive changes associated with sudden gains in PTSD treatments. Cognitive change processes that may be related to sudden gains can be derived from Ehlers and Clark’s (2000) cognitive model of PTSD. This model suggests that excessively negative appraisals of the trauma and/or its sequelae and certain characteristics of the trauma memory (disjointed recall of moments without context information, leading to a “here and now” quality of the memories) play a major role in the maintenance of PTSD. Other cognitive–behavioral models of PTSD have also highlighted appraisal and memory processes (e.g., Brewin, 2014; Foa & Riggs, 1993; Resick & Schnicke, 1993). Prospective studies provided evidence that these processes predict chronic PTSD over and above initial symptom severity (e.g., Beierl, Böllinghaus, Clark, Glucksman, & Ehlers, 2019; Ehring, Ehlers, & Glucksman, 2008). Cognitive therapy for PTSD (CT-PTSD; Ehlers, Clark, Hackmann, McManus, & Fennell, 2005) aims to identify and modify problematic appraisals and elaborate and update the trauma memory with information that gives the worst moments a less threatening meaning. Recent studies of CT-PTSD, prolonged exposure (Foa, Hembree, & Rothbaum, 2007), and cognitive processing therapy (Resick & Schnicke, 1993) provided evidence that changes in PTSD-related appraisals precede symptom change (Kleim et al., 2013; Kumpula et al., 2017; McLean, Yeh, Rosenfield, & Foa, 2015; Schumm, Dickstein, Walter, Owens, & Chard, 2015; Zalta et al., 2014). It has also been shown that the experienced nowness, distress, and vividness of intrusive memories decrease during CT-PTSD (e.g., Hackmann, Ehlers, Speckens, & Clark, 2004). Investigating these maintaining factors in greater detail may help to better understand the processes involved in the occurrence of sudden gains.

Results regarding baseline predictors of sudden gains are inconsistent. Some studies found that higher quality of life and the absence of comorbidity were predictive of sudden gains in depression (Lemmens, DeRubeis, Arntz, Peeters, & Huibers, 2016), or younger age in PTSD (Jun et al., 2013), whereas others found no baseline predictors (e.g., Aderka et al., 2011, 2012; Hunnicutt-Ferguson, Hoxha, & Gollan, 2012).

The present study investigated sudden gains in two large clinical samples of patients with PTSD treated with CT-PTSD in routine clinical care, using the same criteria and including a matched control group. The first aim was to replicate findings that patients who experience a sudden gain during therapy report better outcomes at the end of treatment and at follow-up compared with all patients who did not experience a sudden gain (Hypothesis 1). The second aim was to investigate processes associated with the occurrence of sudden gains. We hypothesized that compared with matched patients who did not experience a sudden gain, patients with sudden gains would show a greater change in negative appraisals and memory characteristics during the sudden gain (Hypothesis 2) and greater change in negative appraisals and memory characteristics before the sudden gain (Hypothesis 3). Baseline predictors of sudden gains and group differences in changes in cognitive processes after the sudden gains were also explored.

## Method

### Participants

This study is a secondary analysis of data drawn from studies investigating the effectiveness of CT-PTSD in routine clinical care. Two cohorts of consecutive patients with PTSD treated in a specialist outpatient clinic for anxiety disorders serving an inner-city population characterized by above-average rates of social deprivation and crime and a greater proportion of ethnic minorities than the national average were treated with CT-PTSD. Patients met the criteria for PTSD according to the Structured Clinical Interview for *DSM-IV* (SCID; First, Gibbon, Spitzer, Williams, & Benjamin, 1997), and PTSD was their main problem. The SCID was administered by trained clinical psychologists. Outcome measures (pretreatment and last-session symptom scores) were available for all patients, including dropouts (14% and 16% respectively), and results are reported by Ehlers et al. (2013) for Sample 1 (*N* = 330) and by Ehlers et al. (2020) for Sample 2 (*N* = 343), see Appendix for data transparency statement. Ethical approval was granted by the local research ethics committee.

The present study included the patients from these consecutive cohorts who provided sufficient week-to-week data to apply Tang and DeRubeis’s (1999) sudden-gains criteria—that is, at least two of three scores prior to a potential gain must be present, as well as at least two of three scores following a potential gain (Sample 1, *N* = 248; Sample 2, *N* = 234). Patient characteristics for each sample are presented in Table 1.[Table-anchor tbl1]

### Treatment

Patients received a course of CT-PTSD (Ehlers et al., 2005) based on Ehlers and Clark’s (2000) cognitive model of PTSD. The treatment aims to reduce the patient’s sense of current threat by changing problematic meanings of the trauma and its consequences; elaborating and updating the memories of the trauma with information that gives them a less threatening meaning at present; discriminating triggers of intrusive memories; and changing behaviors and cognitive processes that maintain PTSD, such as rumination and safety behaviors.

Core interventions in CT-PTSD are as follows: (a) In *individualized case formulation*, the therapist and client collaboratively develop an individualized version of Ehlers and Clark’s (2000) model of PTSD. Treatment procedures are tailored to the formulation. (b) *Reclaiming/rebuilding your life assignments* are designed from the first session onward to address the client’s perceived permanent change after trauma and involve reclaiming or rebuilding activities and social contacts. (c) *Changing problematic appraisals* of the traumas and their sequelae involves information, guided discovery, and behavioral experiments throughout treatment. For appraisals of the traumas, this is closely integrated with the updating-memories procedure. (d) *Updating trauma memories* is a three-step procedure that includes (i) accessing memories of the worst moments during the traumatic events and their threatening meanings, (ii) identifying information that updates these meanings, and (iii) linking the new meanings to the worst moments in memory. (e) *Discrimination training with triggers of reexperiencing* involves systematically spotting idiosyncratic triggers and learning to discriminate between “now” (cues in a new, safe context) and “then” (cue in the traumatic event). (f) *A site visit* completes the memory updating and trigger discrimination. (g) *Dropping unhelpful behaviors and cognitive processes* commonly includes discussing their advantages and disadvantages and behavioral experiments, in which the patient experiments with reducing safety behaviors. (h) A *blueprint* summarizes what the client has learned in treatment and includes plans for any setbacks. Throughout treatment, the work on appraisals is closely interwoven with memory work and is tailored to the case formulation. The specific cognitive therapy techniques will depend on the client’s pattern of emotions and underlying cognitive themes. For further details of treatment procedures, see https://oxcadatresources.com.

Therapists were qualified (i.e., had completed their professional training in clinical psychology, psychiatry, or as a nurse therapist and were registered health professionals) or trainees in these professions. Therapists received training in CT-PTSD (a 2-day workshop followed by case supervision) and attended weekly supervision throughout the studies to ensure treatment fidelity.

The number of sessions depended on the number of traumas and comorbidities to be addressed, usually up to 12 weekly sessions if treatment addressed one or two index traumas and up to 24 sessions if treatment addressed more than two traumas. On average, patients received a mean (*M*) of 11.55 (standard deviation [*SD*] = 4.63) weekly treatment sessions in Sample 1 and a mean of 10.81 (*SD* = 4.35) sessions in Sample 2. If patients were taking psychotropic medication, they had to be on a stable dose for at least 1 month before starting therapy and were asked to stay on that dose for the duration of the treatment.

### Measures

Patients completed the following measures of established reliability and validity at the beginning of every treatment session. Two thirds also completed symptom measures at follow-up (*M* = 280 days after treatment). The measures for Study 2 assessed the same concepts as Study 1 but were updated due to a change in clinic procedures.

#### PTSD symptoms

Both samples completed the Posttraumatic Diagnostic Scale (PDS; Foa, Cashman, Jaycox, & Perry, 1997) to assess PTSD symptom severity. The PDS is a reliable and validated 17-item self-report measure of the PTSD symptoms (Foa et al., 1997) specified in the *DSM-IV* (American Psychiatric Association, 2000). Patients rated the extent to which they were bothered by each of the 17 symptoms during the last week on a scale from 0 (*Not at all*) to 3 (*5 or more times a week/almost always*). The internal consistency at baseline was Cronbach’s α = .85 in Sample 1 and α = .89 in Sample 2. A cutoff of 18 has been found to best predict a PTSD diagnosis (Ehring, Kleim, Clark, Foa, & Ehlers, 2007). Independent ratings of PTSD symptoms were also conducted by trained clinicians experienced in diagnosing PTSD for a subsample using the PTSD Symptom Scale Interview (PSS-I; Foa, Riggs, Dancu, & Rothbaum, 1993) at the beginning and end of treatment. The internal consistency at baseline was Cronbach’s α = .83 in Sample 1 and α = .89 in Sample 2.

#### Depression symptoms

To assess the severity of depressive symptoms, Sample 1 completed the Beck Depression Inventory (BDI; Beck & Steer, 1993a), a 21-item self-report measure with high reliability and validity, Cronbach’s α at baseline = .90. A score of 17 or above indicates moderate depression, and a score of 30 or above indicates severe depression. Sample 2 completed the reliable and validated Patient Health Questionnaire–9 (PHQ-9; Kroenke, Spitzer, & Williams, 2001), Cronbach’s α at baseline = .91. A score of 10 or above suggests a diagnosis of depression.

#### Anxiety symptoms

To assess the severity of anxiety symptoms, Sample 1 completed the Beck Anxiety Inventory (BAI; Beck & Steer, 1993b), a 21-item self-report measure of anxiety symptoms with high reliability and validity, Cronbach’s α at baseline = .93. Sample 2 completed the Generalized Anxiety Disorder 7-item scale (GAD-7; Spitzer, Kroenke, Williams, & Löwe, 2006). A score of 16 or above indicates moderate anxiety, and a score of 26 or above indicates severe anxiety. The internal consistency of the GAD-7 at baseline was Cronbach’s α = .90. A score of 8 or above suggests clinical anxiety.

#### Negative trauma-related appraisals

Patients completed short versions of the Posttraumatic Cognitions Inventory (PTCI; Foa, Ehlers, Clark, Tolin, & Orsillo, 1999). This self-report measure of negative appraisals asks respondents to indicate their agreement with statements indicating negative appraisals about the self, others, and self-blame that are characteristic of patients with PTSD on a scale from 1 (*totally disagree*) to 7 (*totally agree*). Sample 1 completed a 22-item version (PTCI-22; see Kleim et al., 2013), Cronbach’s α at baseline = .92, and Sample 2 completed a revised 20-item version (PTCI-20; Ehlers, Beierl, Ehring, et al., 2019), Cronbach’s α at baseline = .91. The short versions were developed from the items that had the highest factor loadings and best discrimination between people with and without PTSD.

#### Memory characteristics

Patients reported the degree of flashback-like qualities of their unwanted memories of the trauma on the Intrusions Questionnaire (Hackmann et al., 2004). Sample 1 completed a 4-item version of the scale (MEM-4) and reported the degree of perceived nowness, disjointedness, vividness, and distress of their main intrusions, each on a scale between 0 (*Not at all*) and 100 (*Very strongly*; Cronbach’s α at baseline = .62). Sample 2 completed a revised 5-item version (MEM-5; Cronbach’s α at baseline = .84, Ehlers, Beierl, Böllinghaus, et al., 2019) that contained one further item about easy triggering of intrusive memories by many different cues from a study by Halligan, Michael, Clark, and Ehlers (2003).

### Data Analyses

#### Identification of sudden gains

Sudden gains were based on patient scores on the PDS and were defined following the three criteria described by Tang and DeRubeis (1999). The R package suddengains (Version 0.4.0; Wiedemann, Thew, Stott, & Ehlers, 2018) was used to identify sudden gains in both samples. We included PDS scores from the baseline assessment and 12 weekly scores to identify sudden gains between Sessions 2 and 10. Following previous PTSD sudden-gains studies (Doane et al., 2010; Jun et al., 2013; Krüger et al., 2014) a cutoff value for the first criterion was defined as the standard error of the difference from the reliable change index (RCI; Jacobson & Truax, 1991) as calculated by Foa, Zoellner, Feeny, Hembree, and Alvarez-Conrad (2002),^1^ resulting in a cutoff value of 6.15 on the PDS for both samples. A sudden gain was identified between Session *N* (pregain session) and Session *N* + 1 (postgain session) according to the following three criteria:

1 The decrease between two consecutive scores on the PDS was at least 6.15 (PDS_*N*_ – PDS_*N* + 1_ ≥ 6.15). This change represents 12.10% of the total range on the PDS (0–51).
2 PDS scores decreased by at least 25% relative to the pregain score (PDS_*N*_ – PDS_*N* + 1_ ≥ 0.25 × PDS_*N*_).
3 The pooled standard deviation between the mean PDS score of three sessions (or two sessions if three were not available) before the sudden gain (Sessions *N* – 2, *N* – 1, and *N*) and after the sudden gain (Sessions *N* + 1, *N* + 2, and *N* + 3) was greater than the following critical values, which were adjusted for missingness based on *t* values from the two-sample *t* test: *t*_(4;97.5%)_ > 2.776; *t*_(3;97.5%)_ > 3.182; *t*_(2;97.5%)_ > 4.303.

If patients experienced more than one sudden gain, the earliest gain was selected for all further analyses. The stability of gains was assessed in two ways. Following Tang and DeRubeis (1999), a sudden gain was coded as reversed when at least 50% of the magnitude of the sudden gain was lost at any point later in treatment. Following Wucherpfennig, Rubel, Hofmann, et al. (2017), a stable reversal was coded when a reversal also met the criteria for a sudden loss. Sudden losses are defined as the inverse criteria of sudden gains (i.e., parallel criteria to sudden gains for symptom deterioration).

#### Matching procedure

Mahalanobis distance matching, including the propensity score, was used to select matched patients without sudden gains. This method reduces the group differences between patients with and without sudden gains while selecting pairs of patients who are similar based on a list of covariates (Rosenbaum & Rubin, 1985). The following 10 variables were selected as covariates for the matching procedure: age; gender (male; female); months since the main index trauma; type of trauma (interpersonal violence; accidents or disasters; harm to others; other); comorbid depression (yes; no); and baseline scores of PTSD, depression, and anxiety symptoms and negative appraisals and memory characteristics. Propensity scores were calculated using logistic regression with sudden-gain status (yes; no) as the dependent variable and all selected covariates as predictors. Following recommendations by Rosenbaum and Rubin (1983) and Stuart (2010), a 1:1 matching approach was used. Patients were matched on the Mahalanobis distance within calipers of 0.25 to decrease the within-pair differences (Rosenbaum & Rubin, 1985). The R package MatchIt (Version 3.0.2; Ho, Imai, King, & Stuart, 2011) was used to perform the matching. Each matched patient was assigned a “matched session” with the same pregain session number as the sudden-gains patient they were matched with. Two data sets were created for each sample: (a) a “by person” data set including all patients with sufficient week-to-week data (Sample 1: *n* = 248; Sample 2: *n* = 234) and (b) a “matched” data set including all patients with sudden gains and matched patients without sudden gains (Sample 1: *n* = 152; Sample 2: *n* = 174).

#### Statistical analysis

All analyses were performed in R (Version 3.5.2; R Core Team, 2017) through RStudio IDE (Version 1.1.463; RStudio Team, 2018). A significance criterion of α = .05 was set for all analyses. All linear mixed-effect models were estimated using the R package nlme (Version 3.1.137; Pinheiro, Bates, DebRoy, Sarkar, & R Core Team, 2017) with the maximum-likelihood (ML) estimator. The R code for all analyses can be found at https://osf.io/dgt8x/.

The relationship between sudden gains and primary (PTSD symptoms) and secondary (depression and anxiety symptoms) treatment outcomes were analyzed by fitting linear mixed-effect models to account for repeated measures over time. To estimate the effect of the sudden gains at the end of treatment and follow-up, time (categorical), group (all patients with sudden gains and all patients without sudden gains), and the interaction between time and group were included as fixed effects. Baseline scores of the dependent variable were entered as a covariate. Random intercepts were estimated to account for measurements taken from the same individual. Contrasts were specified to test for the effect of sudden gains on the primary outcome. Cohen’s *d* was computed as a standardized effect size of sudden gains on treatment outcome by dividing the adjusted mean difference by the pooled standard deviation at baseline.

Univariate logistic regression models were used to test whether patient characteristics (age, gender, and months since trauma), baseline psychopathology (PTSD symptoms, depression symptoms, anxiety symptoms, and diagnosis of comorbid depression), or baseline cognitive processes (negative appraisals, memory characteristics) showed an association with the occurrence of sudden gains. To test the overall predictive effect of all predictors, multivariate logistic regression models were run. The assumption of linearity of the logit was met for all continuous variables.

Differences in changes in the process variables before, during, and after sudden-gains/matched sessions between the groups were analyzed, fitting one linear mixed-effect model for each process variable using the matched data sets. For all variables, five scores around the sudden gain (*N* – 2, *N* – 1, *N*, *N* + 1, *N* + 2) were extracted to investigate changes in four between-session intervals around the sudden gain (*N* – 2 to *N* – 1, *N* – 1 to *N*, *N* to *N* + 1, *N* + 1 to *N* + 2). The model included the scores of the process variable as the dependent variable and time (*N* – 2, *N* – 1, *N*, *N* + 1, *N* + 2) and group (all patients with sudden gains and all matched patients) as fixed effects. Time was treated as a categorical variable to allow maximum flexibility in the way the outcome changed over time. This approach allowed us to estimate the change in outcome between any two sessions. The interaction between time and group was modeled as a fixed factor to allow the estimation of the difference between groups in the change in outcome for each interval. Random intercepts were estimated to account for measurements taken from the same individual. Contrasts were specified to test for within- and between-groups differences in changes in the process variables during the time intervals around the sudden gain. Estimates of differences between the time intervals within the sudden-gains group are labeled as δ_1_, and those within the matched control group are labeled as δ_2_. The estimates of the difference between the two groups are labeled as Δ_3_. The assumption of normality of the residuals was confirmed visually for all outcomes.

The estimates of the group differences from both samples were meta-analyzed to obtain pooled estimates of the changes in process variables for each analyzed time interval around the sudden gain. Individuals were assumed to be drawn from the same population. Therefore, a fixed-effects model was run using the R package metafor (Version 2.0.0; Viechtbauer, 2010) to estimate the pooled effect based on the adjusted standardized mean difference (SMD; Hedges & Olkin, 1985). The SMD^2^ was calculated based on the estimated difference within the sudden-gains group (δ_1_) and the matched group (δ_2_). The standard deviation for the sudden-gains group and the matched control group was calculated from the difference scores of the investigated interval using the raw data. The *n* was based on the number of patients for which a difference score was available for the investigated interval.

## Results

### Frequency and Characteristics of Sudden Gains

A total of 1,459 and 1,254 between-session intervals were investigated for sudden gains in Samples 1 and 2, respectively. Following the three criteria by Tang and DeRubeis (1999), 76 out of 248 patients (30.65%) experienced a total of 83 sudden gains between Sessions 2 and 10 (median = 5, mode = 3) in Sample 1. In Sample 2, 87 out of 234 patients (37.18%) experienced a total of 100 sudden gains between Sessions 2 and 10 (median = 3, mode = 2). The distribution of the pregain session numbers is presented in Figure S1a in the online supplemental material and showed that sudden gains tended to occur earlier in Sample 2 compared with Sample 1. This may be related to the fact that a core treatment procedure, updating trauma memories, was on average conducted earlier in treatment in the second cohort, in line with guidance by the treatment developers (see Figure S1b in the online supplemental material).

Multiple gains were experienced by 6 patients (2.42%) in Sample 1 (5 patients experienced two sudden gains; 1 patient experienced three sudden gains) and 11 patients (4.70%) in Sample 2 (9 patients experienced two sudden gains; 2 patients experienced three sudden gains). In total, 13 sudden gainers (17.11%) lost 50% of the improvement made during the sudden gain at some point later in treatment (Tang & DeRubeis, 1999) in Sample 1, and 10 (11.49%) in Sample 2, but most of these (92.11% in Sample 1 and 96.55% in Sample 2) regained the improvement made during the sudden gain by the end of treatment. No sudden gainer in Sample 1 and 3 sudden gainers (3.45%) in Sample 2 experienced a stable reversal (see Wucherpfennig, Rubel, Hofmann, et al., 2017).^3^ The average sudden gain was *M* = 12.30 (*SD* = 4.44) points on the PDS in Sample 1 and *M* = 12.11 (*SD* = 3.83) in Sample 2.

### Sudden Gains and Treatment Outcomes

In both samples, patients with sudden gains reported significantly lower PTSD, depression, and anxiety symptoms at the end of treatment than patients without sudden gains (see Table 2). The same result was found for the two subsamples of patients in each cohort (*n*_S1_ = 79, *n*_S2_ = 80) for whom interviewer-assessed PTSD symptoms (PSS-I) were obtained. The differential effect in outcomes between the groups remained significant at follow-up. The mean PTSD symptom severity for patients with and without sudden gains at baseline, the end of therapy, and follow-up is illustrated in the online supplemental material (see Figure S2).[Table-anchor tbl2]

### Baseline Predictors of Sudden Gains

In Sample 1, the multivariate logistic regression model including only statistically significant predictors of the univariate models suggests that higher age and the absence of comorbid major depression predicted the occurrence of sudden gains (see online supplemental material, Table S1 legend). For age, the odds of experiencing a sudden gain increased by a factor of 1.03, 95% confidence interval [CI: 1.01, 1.06], for each year increase in age. For patients with comorbid major depression, the odds of experiencing a sudden gain were 0.45, 95% CI [0.24, 0.81]. However, these results did not replicate in Sample 2. Results from explorative analyses suggested that the association with age in Sample 1 might be driven by three outliers in the sudden-gains group aged around 80 years (see online supplemental material, Figure S3a). In Sample 2, no significant baseline predictors of sudden gains were found. See Table S1 in the online supplemental material for detailed results of the univariate and multivariate logistic regression models investigating baseline predictors of sudden gains.

### Cognitive Processes Associated With Sudden Gains

**Figure 1 fig1:**
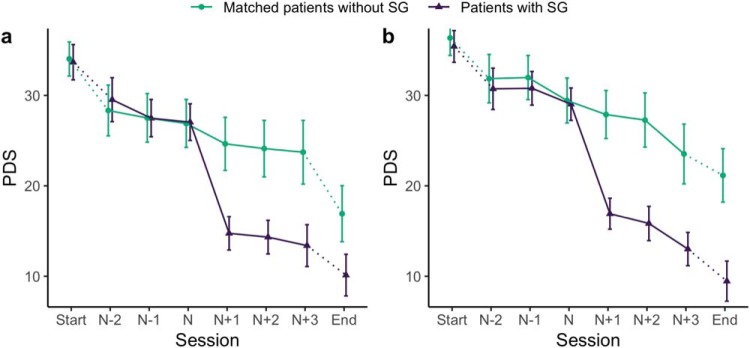
Average change in posttraumatic stress disorder (PTSD) symptoms (Posttraumatic Diagnostic Scale [PDS]) around the sudden-gain/matched session for both samples (Sample 1 [a]: matched patients without sudden gain = 76, patients with sudden gains = 76, total *n* = 152; Sample 2 [b]: matched patients without sudden gain = 87, patients with sudden gains = 87, total *n* = 174). The error bars represent the 95% confidence interval (CI). SG = sudden gain.

When analyzing processes around sudden gains, patients with sudden gains were compared with matched patients who were similar in relevant patient characteristics, symptoms, and cognitive processes at baseline. Figure 1 illustrates that the PTSD-symptom trajectory was very similar for both groups in both samples up to the session before the sudden-gain/matched session and different afterward. All baseline variables were well balanced between the sudden-gains and matched groups for all continuous variables, mean differences in PDS baseline scores (Sample 1 = 0.36, Sample 2 = 0.95), mean difference in months since main index trauma (Sample 1 = 0.64, Sample 2 = 1.64), mean differences in treatment length (Sample 1 = 0.18, Sample 2 = 0.40 sessions), and identical for all categorical variables. Figure 1 shows the average change in PTSD symptoms around the sudden-gain/matched session for both samples. The average sudden gain represented a marked change from the otherwise similar symptom trajectory in the two groups up to the point of the sudden gain. Explorative analyses suggest that both groups in both samples showed a similar degree of improvement from the postgain session or the corresponding matched session to the end of therapy. Baseline correlations between PTSD, depression, and anxiety symptoms, as well as cognitive process measures, were medium to high and statistically significant (see online supplemental material, Table S2).[Fig-anchor fig1]

**Figure 2 fig2:**
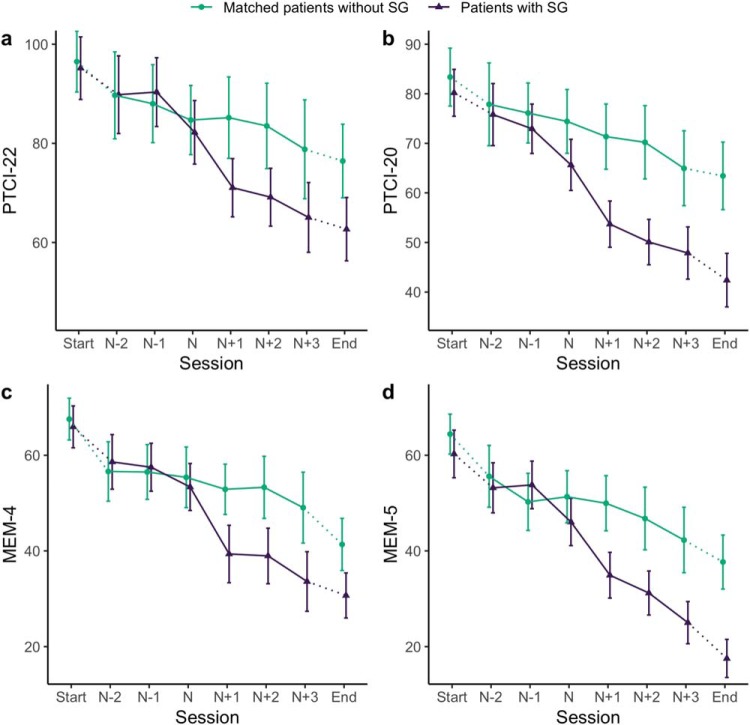
Average change in negative appraisals (Sample 1 [a]: 22-item version of the Posttraumatic Cognitions Inventory [PTCI-22]; Sample 2 [b]: 20-item version of the Posttraumatic Cognitions Inventory [PTCI-20]) and memory characteristics (Sample 1 [c]: 4-item version of the Intrusions Questionnaire scale [MEM-4]; Sample 2 [d]: 5-item version of the Intrusions Questionnaire scale [MEM-5]) around the sudden-gain/matched session for both matched samples (Sample 1: matched patients without sudden gain = 76, patients with sudden gains = 76, total *n* = 152; Sample 2: matched patients without sudden gain = 87, patients with sudden gains = 87, total *n* = 174). The error bars represent the 95% confidence interval [CI]. SG = sudden gain.

**Figure 3 fig3:**
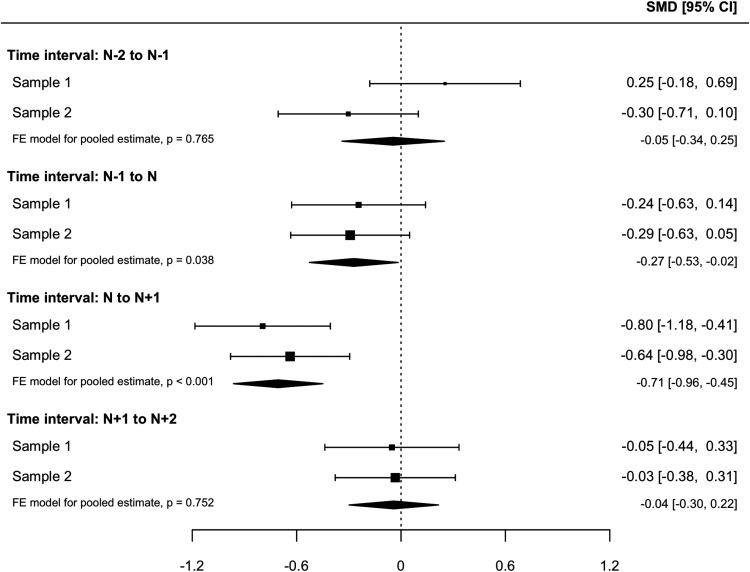
Forest plot of the group difference in changes in negative appraisals (Sample 1: 22-item version of the Posttraumatic Cognitions Inventory [PTCI-22]; Sample 2: 20-item version of the Posttraumatic Cognitions Inventory [PTCI-20]) for time intervals around the sudden gain. Negative numbers indicate greater change in the sudden-gains group; positive numbers indicate greater change in the matched patients without sudden gains. The point sizes are proportional to the precision of the estimates. SMD = standardized mean difference; FE = fixed effect.

**Figure 4 fig4:**
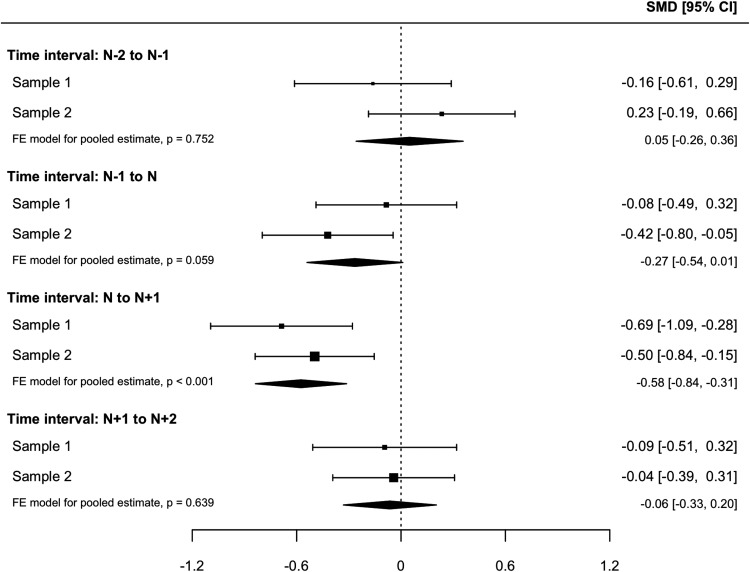
Forest plot of the group difference in changes in memory characteristics (Sample 1: 4-item version of the Intrusions Questionnaire scale [MEM-4]; Sample 2: 5-item version of the Intrusions Questionnaire scale [MEM-5]) for time intervals around the sudden gain. Negative numbers indicate greater change in the sudden gains group; positive numbers indicate greater change in the matched patients without sudden gains. The point sizes are proportional to the precision of the estimates. SMD = standardized mean difference; FE = fixed effect.

Figure 2 shows the average change in negative appraisals and memory characteristics around the sudden-gain/matched session. Within- and between-group changes are presented in Table 3. During the sudden gain (*N* to *N* + 1), in both samples, the sudden-gains group showed large and statistically significant decreases in cognitive processes, which were larger than those observed in the matched control group for both negative appraisals and memory characteristics. The pooled estimates of Samples 1 and 2 for change in negative appraisals (β = −0.71, 95% CI [−0.96, −0.45], *p* < .001) and memory characteristics (β = −0.58, 95% CI [−0.84, −0.31], *p* < .001) during sudden gains showed significant differences between the sudden-gains and matched groups (Figures 3 and 4). [Fig-anchor fig2][Table-anchor tbl3][Fig-anchor fig3][Fig-anchor fig4]

In the interval before the sudden gain (*N* – 1 to N), negative appraisals already showed decreases in the sudden gains group for both samples, whereas the decreases in the matched groups were nonsignificant. For memory characteristics, a significant decrease in the sudden-gains group was found in Sample 2 and a trend in Sample 1, whereas the decreases in matched controls were nonsignificant. The pooled estimates for the group differences in change in cognitive processes preceding sudden gains showed a significant group difference for negative appraisals, β = −0.27, 95% CI [−0.53, −0.02], *p* = .038, and the same effect size, but that was not statistically significant for memory characteristics, β = −0.27, 95% CI [−0.54, 0.01], *p* = .059, indicating greater cognitive change in the sudden-gains group (Figures 3 and 4) before the sudden gain. For the postgain session, no significant changes were found in negative appraisals or memory characteristics for either group in either sample.

## Discussion

This study investigated change processes around sudden gains during an empirically validated treatment for PTSD in routine clinical practice in two samples of consecutive cases and found that a substantial subgroup of around one third of patients showed large improvements in PTSD symptoms from one session to the next. In line with the first hypothesis, sudden gains were associated with better treatment outcomes in both samples, as measured by both self-reported and interviewer-rated PTSD-symptom severity. This replicates previous findings with other psychological therapies for PTSD (e.g., Aderka et al., 2011; Kelly et al., 2009; König et al., 2014; Krüger et al., 2014). To analyze change processes around sudden gains, this study compared changes between patients with sudden gains and matched patients without sudden gains. In line with the second hypothesis, patients who experienced a sudden gain in PTSD symptoms showed large concurrent improvements in cognitive processes thought to maintain PTSD (negative appraisals and memory characteristics; Ehlers & Clark, 2000). In line with the third hypothesis, pooled estimates across both samples showed that negative appraisals had already decreased in the session prior to sudden gains to a larger extent than for matched patients before the corresponding matched session, and there was also a trend for a greater decrease in trauma memory characteristics.

Sudden gains occurred in a similar proportion of patients in both samples (30.65% and 37.18%), with a similar average magnitude of the sudden gain (*M* = 12.30, *SD* = 4.44 and *M* = 12.11, *SD* = 3.83). These results are similar to previous studies in PTSD (e.g., König et al., 2014, 22%; Krüger et al., 2014, 25%) and other disorders (Aderka et al., 2012, 37%). Although a minority of patients with sudden gains met the Tang and DeRubeis (1999) criterion for a subsequent loss of 50% of the gain (reversal), most of these regained the improvements made during the sudden gain by the end of therapy, suggesting that reversals were mainly temporary deteriorations. Only three sudden gainers in Sample 2 experienced a stable reversal that met the criteria for a sudden loss. There was an interesting difference between the samples in that more patients experienced sudden gains early in treatment in Sample 2 compared with Sample 1 (see online supplemental material, Figure S1), which paralleled the earlier use of the updating-memory procedure in Sample 2. This might indicate that starting to work on the trauma memory early in treatment facilitates large improvements in some patients.

No evidence for consistent baseline predictors of sudden gains was found across the samples. In contrast to Vittengl, Clark, and Jarrett (2005), we did not find that the baseline severity of the sudden-gains outcome measure (PDS) predicts sudden gains in PTSD. Similar to other studies (e.g., Hunnicutt-Ferguson et al., 2012; Vittengl et al., 2005), we did not find evidence that cognitive processes at the beginning of treatment predict the occurrence of sudden gains, suggesting that processes during therapy are more important in the occurrence of sudden gains than patient characteristics or symptomatology before the treatment.

In line with some other sudden-gains studies in depression (Tang & DeRubeis, 1999; Tang et al., 2005), this study also found evidence for cognitive changes prior to the sudden gain (see Table 3, δ_1_ for negative appraisals from *N* – 1 to *N*). However, matched patients without sudden gains also experienced nonsignificant decreases. This highlights the importance of a control group when analyzing processes around sudden gains. Although the observed group differences with effect sizes of −0.24 and −0.29 did not reach significance within each sample, the meta-analysis suggested greater changes in appraisals in the sudden-gains groups, −0.27, 95% CI [−0.53, −0.02], *p* = .038 (see Figure 3). Similar effects for group differences were obtained for memory characteristics, with a pooled estimate of −0.27, which was not statistically significant. Thus, there was some support for Hypothesis 3, although the effects were small. Three other studies did not find evidence for significant cognitive changes preceding sudden gains in individual samples of other disorders (Andrusyna et al., 2006; Bohn et al., 2013; Hofmann et al., 2006), suggesting overall small effects. Larger samples or pooling data across samples may be a way to further investigate the effect we found in this study. The observation that PTSD symptoms and cognitive-process variables are correlated with each other at baseline (see online supplemental material, Table S2) does not explain this pattern of findings.

This study also found further evidence for simultaneous changes of cognitive processes with the sudden gain in PTSD symptoms, supporting Hypothesis 2. These findings might partly be explained by the correlations between symptoms and cognitive processes in this sample. Our results show evidence that these concurrent changes are preceded by changes in cognitions.

### Strength and Limitations

This study investigated the processes associated with sudden gains in two large clinical samples of patients with PTSD treated in routine clinical care with an empirically validated psychological treatment who completed weekly symptom and process measures. The large samples allowed for an advanced matching approach to create control groups of similar patients without sudden gains. The statistical modeling approach ensured a detailed analysis of potential process variables leading up to the gain, during the gain, and after the gain. Further, this is the first study of sudden gains to report identifying sudden gains using a fully automated approach and sharing the code publicly. A more detailed discussion of the benefits of transparent research practices and replication studies in the psychological sciences can be found elsewhere (Nosek et al., 2015; Open Science Collaboration, 2015; Tackett et al., 2017).

The limitations of this study include the variations in measures across the samples that reflected changes in clinic procedures. The internal reliability of the measure assessing memory characteristics in Sample 1 was low (MEM-4; Cronbach’s α at baseline = .62). However, because similar results for changes in memory characteristics in Sample 2 were obtained with an improved measure (MEM-5, Cronbach’s α at baseline = .84), the findings appear to be valid. However, the measure only contained one item measuring the disjointedness of memories and did not assess other potentially relevant aspects of memory disorganization, so the effect may have been underestimated. In addition, all measures assessing changes around sudden gains were patient self-reports, and other data, such as ratings of videotapes, were not available. Furthermore, the standard criteria used to identify sudden gains may yield some false positives. In a data simulation study, Vittengl, Clark, Thase, and Jarrett (2015) found that some sudden gains are due to random symptom fluctuation during therapy. Thomas and Persons (2013) argue that some sudden gains represent the largest and most stable change occurring in a gradual course of change.

### Conclusions

This study showed, in two independent, consecutive samples, that sudden gains occur in about a third of patients treated with CT-PTSD and reliably predict better treatment outcomes. There were no reliable baseline predictors of sudden gains, suggesting that they can occur in a wide range of patients. When sudden gains occur, they are associated with broad changes in cognitive processes. Furthermore, there was some evidence that sudden gains in PTSD symptoms are preceded by a change in key variables from Ehlers and Clark’s (2000) cognitive model of PTSD. This finding supports the role of changes in appraisals and memory characteristics in improving PTSD symptoms with cognitive–behavioral therapy, as suggested by several theoretical models (e.g., Brewin, 2014; Ehlers & Clark, 2000; Foa & Riggs, 1993; Resick & Schnicke, 1993). If the processes identified in this study replicate in future sudden-gains studies, the results could indicate the importance of maximizing cognitive change to promote symptom change in PTSD. This could be achieved by focusing early in therapy on the individual meanings of the trauma that lead to a sense of current threat. The updating-memories procedure used in the cohort studies for this purpose was associated with sudden gains in therapy as early as Session 2 (see online supplemental material, Figure S1b). Future research is also needed to test these processes in different treatment approaches, for example, more exposure-based treatments.

From a methodological perspective, the present results highlight the importance of a control group when analyzing processes associated with sudden gains. Whereas this and other studies (e.g., Wucherpfennig, Rubel, Hofmann, et al., 2017) assigned matched sessions based on the pregain session of the matched sudden-gains patient, alternative methods also need to be explored. For example, taking the session with the largest gain in patients without sudden gains as the matched session may be a sensible alternative when analyzing processes around sudden gains. Smaller intervals of measuring symptom and process variables would allow a more accurate identification of the point during the week at which the sudden gains occurred and also the identification of processes that precede and follow the gain more closely in time.

These findings provide a better understanding of how CT-PTSD works, especially in patients with sudden gains. Further research needs to investigate whether certain therapeutic techniques or general therapeutic processes play a role in creating and maintaining sudden symptom improvements.

## Supplementary Material

10.1037/ccp0000488.supp

## Figures and Tables

**Table 1 tbl1:** Patient Characteristics for Both Samples

	Sample 1 (*n* = 248)			Sample 2 (*n* = 234)		
Variable	*n*	%	*M* (*SD*)	*n*	%	*M* (*SD*)
Age	248		38.90 (11.23)	234		37.82 (11.14)
Months since main index traumatic event	238		37.61 (57.94)	232		52.34 (78.45)
Weekly treatment sessions	248		11.55 (4.63)	233		10.81 (4.35)
Gender						
Female	105	42.3		103	44.0	
Male	143	57.7		131	56.0	
Relationship						
Married/cohabit	87	35.1		92	39.3	
Divorced/separated/widowed	46	18.5		28	12.0	
Never married	108	43.5		106	45.3	
No information	7	2.8		8	3.4	
Ethnicity						
Black	64	25.8		56	23.9	
Caucasian	138	55.6		150	64.1	
Other	46	18.5		28	12.0	
Education						
University	71	28.6		69	29.5	
A-levels	37	14.9		30	12.8	
GCSE	69	27.8		53	22.6	
Other	54	21.8		37	15.8	
No information	17	6.9		45	19.2	
Employment						
Employed/self-employed	93	37.5		109	46.6	
Student	12	4.8		10	4.3	
Sick leave	34	13.7		13	5.6	
Disability/retired	22	8.9		12	5.1	
Unemployed	73	29.4		76	32.5	
No information	14	5.6		14	6.0	
Type of main traumatic event						
Interpersonal violence	144	58.1		147	62.8	
Accident or disaster	51	20.6		47	20.1	
Death or harm to others	23	9.3		28	12.0	
Other	30	12.1		12	5.1	
Childhood trauma						
No	149	60.1		163	69.7	
Yes	34	13.7		29	12.4	
No information	65	26.2		42	17.9	
Comorbid major depression						
No	124	50.0		111	47.4	
Yes	124	50.0		123	52.6	
*Note*. *n* = number of available responses for each variable; % = percentage of total sample; GCSE = General Certificate of Secondary Education.						

**Table 2 tbl2:** Primary and Secondary Treatment Outcomes for All Patients With and Without Sudden Gains

	Unadjusted mean (*SD*)			Adjusted difference at end of treatment and follow-up^a^			
Measure/Time	*n*	SG	*n*	No SG	Mean [95% CI]	*d* [95% CI]	*p*
PTSD symptoms (self-report, PDS)							
*S*_1_: baseline	76	33.66 (8.66)	172	34.10 (8.72)			
*S*_1_: end	76	10.13 (10.22)	172	19.25 (14.47)	−8.81 [−10.40, −7.22]	1.02 [0.73, 1.30]	<.001
*S*_1_: FU	67	11.27 (10.09)	105	16.51 (12.94)	−6.54 [−8.22, −4.86]	0.75 [0.44, 1.07]	<.001
*S*_2_: baseline	84	35.41 (8.22)	142	34.04 (10.33)			
*S*_2_: end	87	9.46 (10.54)	147	19.02 (14.00)	−11.04 [−12.60, −9.47]	1.15 [0.86, 1.43]	<.001
*S*_2_: FU	59	10.23 (11.14)	74	17.83 (14.29)	−10.18 [−12.01, −8.36]	1.06 [0.69, 1.42]	<.001
PTSD symptoms (interviewer, PSS-I)							
*S*_1_: baseline	32	31.35 (8.16)	47	30.35 (9.01)			
*S*_1_: end	32	11.09 (11.34)	47	16.78 (14.11)	−6.37 [−11.56, −1.76]	0.70 [0.23, 1.16]	.016
*S*_2_: baseline	42	35.30 (7.11)	38	33.34 (8.48)			
*S*_2_: end	42	8.52 (7.71)	38	15.79 (13.36)	−8.18 [−12.61, −3.75]	0.71 [0.25, 1.16]	<.001
Depression symptoms (*S*_1_: BDI; *S*_2_: PHQ-9)							
*S*_1_: baseline	76	26.29 (12.17)	172	28.06 (11.76)			
*S*_1_: end	76	10.85 (10.68)	172	18.05 (14.14)	−6.12 [−7.61, −4.62]	0.51 [0.24, 0.79]	<.001
*S*_1_: FU	66	11.11 (10.14)	102	14.36 (12.10)	−3.34 [−4.93, −1.76]	0.28 [−0.03, 0.59]	.035
*S*_2_: baseline	85	17.08 (6.40)	142	16.39 (7.25)			
*S*_2_: end	87	4.53 (5.59)	146	10.50 (7.94)	−6.22 [−7.12, −5.32]	0.90 [0.62, 1.17]	<.001
*S*_2_: FU	59	5.94 (6.70)	73	9.99 (8.45)	−4.54 [−5.61, −3.46]	0.65 [0.30, 1.01]	<.001
Anxiety symptoms (*S*_1_: BAI; *S*_2_: GAD-7)							
*S*_1_: baseline	74	25.87 (13.31)	167	29.78 (13.79)			
*S*_1_: end	76	8.07 (9.85)	172	17.11 (15.71)	−6.90 [−8.60, −5.20]	0.50 [0.23, 0.78]	<.001
*S*_1_: FU	67	9.40 (10.94)	102	13.29 (13.53)	−4.18 [−5.97, −2.40]	0.31 [−0.01, 0.61]	.019
*S*_2_: baseline	85	14.46 (5.32)	143	14.16 (5.52)			
*S*_2_: end	87	3.78 (4.23)	145	8.50 (6.76)	−4.83 [−5.59, −4.08]	0.89 [0.61, 1.17]	<.001
*S*_2_: FU	59	5.04 (5.11)	74	8.27 (6.92)	−3.65 [−4.55, −2.75]	0.67 [0.32, 1.02]	<.001
*Note*. SG = sudden gain; *d* = between-group standardized effect size; PTSD = posttraumatic stress disorder; PDS = Posttraumatic Diagnostic Scale; *S*_1_ = Sample 1 (*n* = 248); end = end of treatment; FU = follow-up; *S*_2_ = Sample 2 (*n* = 234); PSS-I = PTSD Symptom Scale Interview; BDI = Beck Depression Inventory; PHQ-9 = Patient Health Questionnaire–9; BAI = Beck Anxiety Inventory; GAD-7 = Generalized Anxiety Disorder 7-item scale.							
^a^ The difference is adjusted for baseline scores.							

**Table 3 tbl3:** Estimated Changes in Negative Appraisals and Memory Characteristics During the Time Intervals Around the Sudden Gain

	Sudden-gains group		Matched group		Group difference	
Measure/Time interval	δ_1_ (*SE*)	*p*	δ_2_ (*SE*)	*p*	Δ_3_(*SE*)	*p*
*S*_1_: Negative appraisals						
*N* – 2 to *N* – 1	1.36 (2.01)	.500	−1.60 (2.09)	.445	2.95 (2.90)	.309
*N* – 1 to *N*	−6.08 (1.85)	.001	−3.03 (1.92)	.114	−3.05 (2.66)	.252
*N* to *N* + 1	−10.90 (1.80)	<.001	−0.39 (1.89)	.837	−10.51 (2.61)	<.001
*N* + 1 to *N* + 2	−3.05 (1.80)	.091	−2.51 (1.98)	.205	−0.53 (2.68)	.842
*S*_2_: Negative appraisals						
*N* – 2 to *N* – 1	−5.14 (2.18)	.018	−0.71 (2.36)	.762	−4.42 (3.22)	.169
*N* – 1 to *N*	−7.40 (1.94)	<.001	−2.89 (2.14)	.176	−4.51 (2.88)	.117
*N* to *N* + 1	−12.12 (1.93)	<.001	−2.90 (2.11)	.169	−9.22 (2.86)	.001
*N* + 1 to *N* + 2	−3.63 (1.92)	.058	−3.16 (2.19)	.150	−0.48 (2.91)	.870
*S*_1_: Memory characteristics						
*N* – 2 to *N* – 1	−2.12 (2.45)	.386	0.25 (2.61)	.924	−2.37 (3.58)	.508
*N* – 1 to *N*	−3.96 (2.28)	.082	−2.60 (2.40)	.278	−1.36 (3.31)	.680
*N* to *N* + 1	−14.18 (2.29)	<.001	−3.63 (2.35)	.122	−10.54 (3.28)	.001
*N* + 1 to *N* + 2	−1.81 (2.43)	.457	−0.41 (2.41)	.864	−1.40 (3.42)	.683
*S*_2_: Memory characteristics						
*N* – 2 to *N* – 1	−1.83 (2.31)	.430	−5.23 (2.54)	.039	3.41 (3.43)	.321
*N* – 1 to *N*	−8.09 (2.06)	<.001	−1.54 (2.29)	.501	−6.56 (3.08)	.033
*N* to *N* + 1	−11.26 (1.98)	<.001	−3.11 (2.11)	.140	−8.15 (2.89)	.005
*N* + 1 to *N* + 2	−3.80 (1.98)	.056	−3.18 (2.20)	.148	−0.62 (2.96)	.835
*Note*. SE = standard error. For each time interval, the estimated changes were compared within (δ_1_, δ_2_) and between (Δ_3_) groups.						

## Footnotes

1 The standard error of the difference was computed using the test–retest reliability (*r_xx_*) of .83 and standard deviation of a nonclinical sample (*s_1_*) of 10.54 of the PDS as reported by Foa et al. (1997). The standard error of measurement for the scale (*S_E_* = 4.35) was computed using $S_{E}=\;s_{1}\;\times \;\sqrt{1\;-\;r_{xx}}$. The standard error of the difference (*S*_diff_ = 6.15) was computed using $S_{\text{diff}}=\;\sqrt{2\;\times \;{\left(S_{E}\right)}^{2}}$ by Foa et al. (2002).

2

$$\begin{array}{rr} \text{SMD} & =\frac{\delta_{1}\;-\;\delta_{2}}{S},\;\;\;\text{where}\\ \text{S} & =\sqrt{\frac{\left(n_{\text{SG}}-1\right)\;\times \;{\text{SD}}_{{\text{diff}}_{1}}^{2}\;+\;\left(n_{\text{Control}}\;-\;1\right)\;\times {\text{SD}}_{{\text{diff}}_{2}}^{2}}{n_{\text{SG}}+n_{\text{Control}}-2}},\;\;\;\text{where}\\ {\text{SD}}_{{\text{diff}}_{1,2}} & =\text{SD}\left(x_{t}-x_{t+1}\right)\;\text{for}\;{\text{SG}}_{\left(1\right)}\;\text{and}\;{\text{Control}}_{\left(2\right)}\;\text{group}. \end{array}$$

3 Following suggestions from a reviewer and the editor, we repeated all analyses after excluding patients who met the criteria for a stable reversal, and the results did not differ.

## Data Transparency

The data sets used in this study constitute subsamples of two data sets of consecutive samples of patients with PTSD treated with CT-PTSD that reported measures at a sufficient number of data points so that sudden gains could be analyzed. Outcome data for the whole samples have been reported in separate articles. MS 1 (published) and MS 2 (under review) presented outcome data for the effectiveness of CT-PTSD, looking primarily at the variables of PTSD symptoms, depression symptoms, and anxiety symptoms in Data Sets 1 and 2, respectively. MS 3 (published) focuses on mediation of clinical improvement investigating the variables of PTSD symptoms and negative appraisals in Data set 1. MS 4 (published) and MS 5 (soon to be submitted) focus on the variables of PTSD symptoms, sleep duration, and sleep quality (not used in the current study) in Data Sets 1 and 2, respectively. MS 6 (the current article) focuses on sudden gains, looking at the variables of PTSD symptoms, depression symptoms, anxiety symptoms, negative appraisals, and memory characteristics in both data sets. MS 7 (soon to be submitted) will focus on mediation of clinical improvement, examining the variables of PTSD symptoms, negative appraisals, memory characteristics, response to intrusions, and safety behaviors in Data Set 2.
