# Supplementary material for: Cognitive Processes Associated With Sudden Gains in Cognitive Therapy for Posttraumatic Stress Disorder in Routine Care
Source: J Consult Clin Psychol. 2020 Mar 5;88(5):455–69. doi: 10.1037/ccp0000488 (PMC7144503; doi:10.1037/ccp0000488)
Supplement: Supplementary file 1 [file sg-ptsd-audit-jccp-supp-rev-3-v2.docx]

**Online Supplemental Material**

Table S1

*Logistic regression analyses predicting the occurrence of sudden gains*

|  | Sample 1: Univariate ^†^ | |  | Sample 1: Multivariate ^†^ | |  | Sample 2: Univariate ^†^ | |  | Sample 2: Multivariate ^†^ | |
| --- | --- | --- | --- | --- | --- | --- | --- | --- | --- | --- | --- |
| Term | OR (95% CI) | *p* |  | OR (95% CI) | *p* |  | OR (95% CI) | *p* |  | OR (95% CI) | *p* |
| Demographic predictors |  |  |  |  |  |  |  |  |  |  |  |
| Age | 1.03 (1.01 to 1.06) | .007 |  | 1.04 (1.01 to 1.07) | .010^‡^ |  | 1.00 (0.98 to 1.02) | .938 |  | 1.00 (0.97 to 1.02) | .817 |
| Gender | 0.94 (0.54 to 1.62) | .819 |  | 1.20 (0.62 to 2.34) | .589 |  | 0.95 (0.56 to 1.62) | .848 |  | 1.10 (0.61 to 2.00) | .742 |
| Months since trauma | 1.00 (0.99 to 1.00) | .135 |  | 1.00 (0.99 to 1.00) | .201 |  | 1.00 (1.00 to 1.00) | .629 |  | 1.00 (1.00 to 1.00) | .918 |
| Baseline psychopathology |  |  |  |  |  |  |  |  |  |  |  |
| PTSD symptoms | 0.99 (0.96 to 1.03) | .712 |  | 1.04 (0.98 to 1.10) | .192 |  | 1.02 (0.99 to 1.05) | .300 |  | 1.05 (0.99 to 1.11) | .110 |
| Depression symptoms | 0.99 (0.96 to 1.01) | .282 |  | 1.00 (0.95 to 1.06) | .911 |  | 1.01 (0.98 to 1.06) | .471 |  | 1.02 (0.93 to 1.11) | .649 |
| Anxiety symptoms | 0.98 (0.96 to 1.00) | .042 |  | 0.97 (0.94 to 1.00) | .076^‡^  |  | 1.01 (0.96 to 1.06) | .688 |  | 0.96 (0.87 to 1.06) | .475 |
| Comorbid depression | 0.39 (0.22 to 0.68) | .001 |  | 0.46 (0.21 to 0.96) | .041^‡^ |  | 1.02 (0.60 to 1.74) | .942 |  | 1.13 (0.58 to 2.24) | .714 |
| Baseline cognitive processes |  |  |  |  |  |  |  |  |  |  |  |
| Negative cognitions | 0.99 (0.98 to 1.00) | .210 |  | 1.00 (0.98 to 1.02) | .979 |  | 1.00 (0.99 to 1.01) | .890 |  | 1.00 (0.98 to 1.01) | .661 |
| Memory characteristics | 0.99 (0.97 to 1.00) | .171 |  | 1.00 (0.98 to 1.02) | .744 |  | 1.00 (0.99 to 1.01) | .908 |  | 0.99 (0.97 to 1.01) | .266 |

*Note.* ^†^ Logistic regression model. Sudden gains (0 = no, 1 = yes). Gender (0 = male, 1 = female). ^‡^ A multivariate model using only the significant predictors from the univariate model gave the following results: Age: OR = 1.03, 95% CI [1.01 to 1.06], *p* = .012; Anxiety symptoms: OR = 0.99, 95% CI [0.96 to 1.01], *p* = .269; Comorbid depression: OR = 0.45, 95% CI [0.24 to 0.81], *p* = .009.

Table S2

*Pearson correlations between symptoms and cognitive processes in Sample 1 and 2 at baseline*

|  | S_1_: PDS | S_1_: PTCI-22 | S_1_: MEM-4 | S_1_: BDI | S_1_: BAI |
| --- | --- | --- | --- | --- | --- |
| S_1_: PDS | - |  |  |  |  |
| S_1_: PTCI-22 | .68 | - |  |  |  |
| S_1_: MEM-4 | .46 | .43 | - |  |  |
| S_1_: BDI | .66 | .77 | .47 | - |  |
| S_1_: BAI | .60 | .57 | .40 | .63 | - |
|  |  |  |  |  |  |
|  | S_2_: PDS | S_2_: PTCI-20 | S_2_: MEM-5 | S_2_: PHQ-9 | S_2_: GAD-7 |
| S_2_: PDS | - |  |  |  |  |
| S_2_: PTCI-20 | .66 | - |  |  |  |
| S_2_: MEM-5 | .62 | .46 | - |  |  |
| S_2_: PHQ-9 | .75 | .58 | .52 | - |  |
| S_2_: GAD-7 | .72 | .58 | .49 | .82 | - |

*Note.* S_1_ = Sample 1 ($n$ = 248). S_1_ = Sample 2 ($n$ = 234). All correlations were statistically significant at *p* < .001.

*Figure S1.* (a) Distribution of pregain sessions in percent for all sudden gains in Sample 1 and 2. Percentages are based on the number of patients who experienced sudden gains in each sample respectively (Sample 1: *n* = 76; Sample 2: *n* = 87). (b) Percentage of sessions early in treatment with updating procedure.*
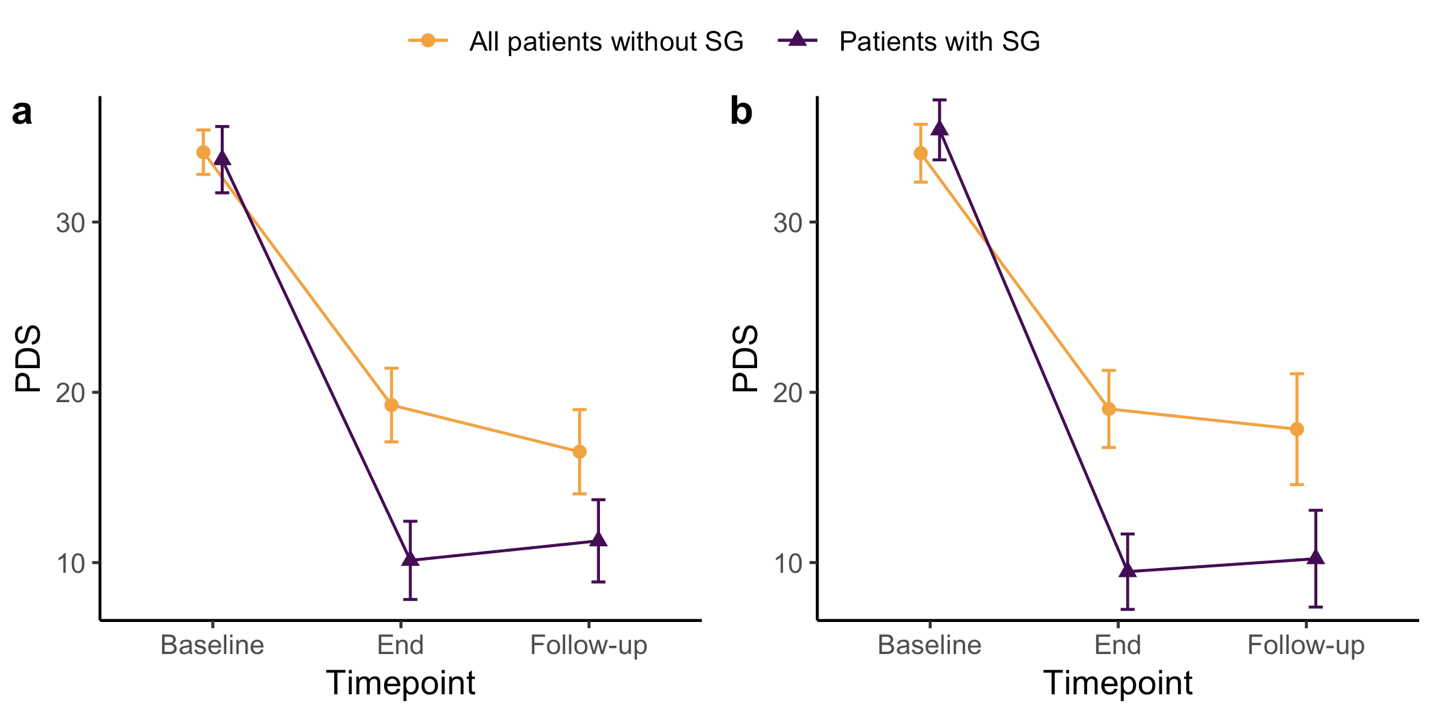
*

*Figure S2.* Mean PTSD severity (PDS) and 95% CI for patients with and without sudden gains for Sample 1 (a), all patients without sudden gains = 172, patients with sudden gain = 76, total *n* = 248, and Sample 2 (b) all patients without sudden gain = 147, patients with SG = 87, total *n* = 234. SG = Sudden gain.

*
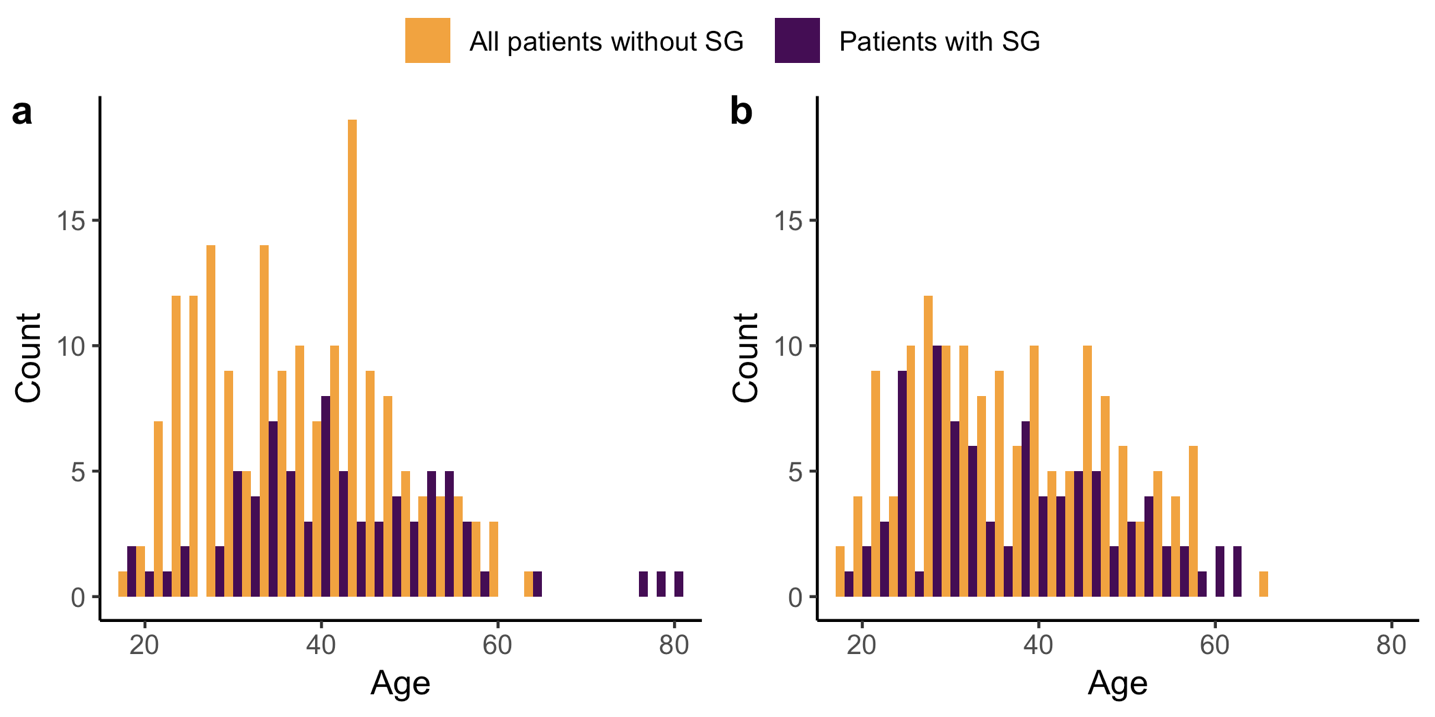
Figure S3*. Age distribution of patients with and all patients without sudden gains in Sample 1 (a) and Sample 2 (b).
